# Case report: The case of T-cell acute lymphoblastic leukemia treated with chemotherapy followed by anti-CD7 CAR-T cells using retroviral vector

**DOI:** 10.3389/fimmu.2024.1519055

**Published:** 2025-01-14

**Authors:** Huanhuan Zhou, Wenxiang Zhu, Qihong Ma, Ning Liu, Mengdi Jin, Yaru Feng, Lijun Zhao, Rui Sun, Rongyou Li, Huaxiu Li, Yuanyuan Shi, Jianxun Wang, Liqiong Liu, Zhi Guo

**Affiliations:** ^1^ Department of Hematology, The Sixth Affiliated Hospital of Shenzhen University Health Science Center, Shenzhen, China; ^2^ Nanozyme Laboratory in Zhongyuan, Henan Academy of Innovations in Medical Science, Zhengzhou, China; ^3^ Shenzhen Cell Valley Biomedical Co., LTD, Shenzhen, China; ^4^ National Health Commission (NHC) Key Laboratory of Nuclear Technology Medical Transformation (Mianyang Central Hospital), Mianyang, China; ^5^ Institute of Infection, Immunology and Tumor Microenvironment, Hubei Province Key Laboratory of Occupational Hazard Identification and Control, School of Medicine, Wuhan University of Science and Technology, Wuhan, China

**Keywords:** T-ALL, CD7 CAR-T, chemotherapy, retroviral vectors, complete remission

## Abstract

CD7-targeted chimeric antigen receptor-T (CAR-T) cell therapy has shown great promise in the treatment of relapsed/refractory T-cell acute lymphoblastic leukemia (T-ALL). In this study, we reported a case of a 34-year-old male patient with T-ALL who finally developed multi-line drug resistance and refractoriness after multiple lines of high-intensity chemotherapy. After physician evaluation, this patient received allogeneic hematopoietic stem cell transplantation (allo-HSCT). Then, The patient remained in complete remission (CR) for four months before a relapse with 26.64% chimerism rate, so he was treated with allogeneic anti-CD7 CAR-T cells after chemotherapy reducing the tumor burden. The CAR-T product was a novel anti-CD7 CAR-T based on retroviral vectors (RV). After infusion, the patient achieved CR within 1 month after anti-CD7 CAR-T infusion and the remission has been ongoing for 9 months to date. Cytokine release syndrome (CRS) 1 was experienced while no immune effector cell-associated neurotoxicity syndrome (ICANS) was found. In addition, CAR copy number peaked at 350, 758 copies/μg on day 6. This case report of clinical treatment of T-ALL with anti-CD7 CAR-T cells prepared using a retroviral vector without gene editing and combined with chemotherapy, which demonstrated that the RV-based anti-CD7 CAR-T cells had good therapeutic effect and high safety in triple-refractory T-ALL patients.

## Introduction

Lymphocytic leukemia (ALL) is a hematological malignancy originating from B or T lymphocytes, and its pathogenesis involves genetic changes such as chromosomal translocation, mutations, and abnormal regulation of the cell cycle (1, 2). The total incidence rate of ALL is 3.85/100000 people, and patients under 19 years old account for 60% of the total ALL patients (3). Among them, acute T-lymphocytic leukemia (T-ALL) accounts for 15% -25% of ALL, and is known as the invasive subtype of ALL (4). T-ALL accounts for about 10% -15% of childhood ALL patients. The incidence rate peaked between 2-5 years old, with a median age of 18 years. Men are more than women. The complete remission (CR) rate is low, and the median survival period is only 11-17 months (4). T-ALL is further divided into early T-cell type (early T-ALL) and mature T-cell type, with the former often having a worse prognosis than the latter (3).

Meanwhile, relapsed/refractory (r/r) T-ALL still faces significant challenges, with extremely poor prognosis and a 3-5 year OS rate of only 7% to 23%. Chimeric antigen receptor T cells refer to T cells that have been genetically modified to express chimeric antigen receptors (CARs) that can specifically recognize tumor cell antigen molecules, thereby obtaining antigen-specific killing ability against tumor cells. CAR-T has achieved significant results in the treatment of hematological tumors and is a promising immunotherapy approach. CAR-T therapy can significantly improve the prognosis of patients with r/r B-ALL (5–7). However, due to the co-expression of many optional target antigens between normal and malignant T cells, CAR-T targeting T cell antigens will clear endogenous T cells in patients, which may lead to severe T cell immune deficiency. Therefore, designing CAR-T treatment plans for T-ALL remains a challenge (8). Nevertheless, researchers are actively seeking specific targets that can be applied to such patients. Some preclinical studies have found that CAR-T targeting CD7 has anti-tumor effects *in vitro* and preclinical mouse model experiments (9–12).

In this study, we prepared novel anti-CD7 CAR-T based on retroviral vector technology. For RV packaging, Phoenix-ECO cells and PG13 cells were used to produce stable PG13 CD7 RV producer cell lines. PG13 was Gibbon Ape Leukemia Virus (GALV) Packaging cell lines. A patient with r/r T-ALL treated with allogeneic hematopoietic stem cell transplantation follow by anti-CD7 CAR-T cell therapy was also reported. One month after treatment, the patient had a complete remission (CR) that had lasted for more than 9 months.

## Case presentation

**Figure 1:**
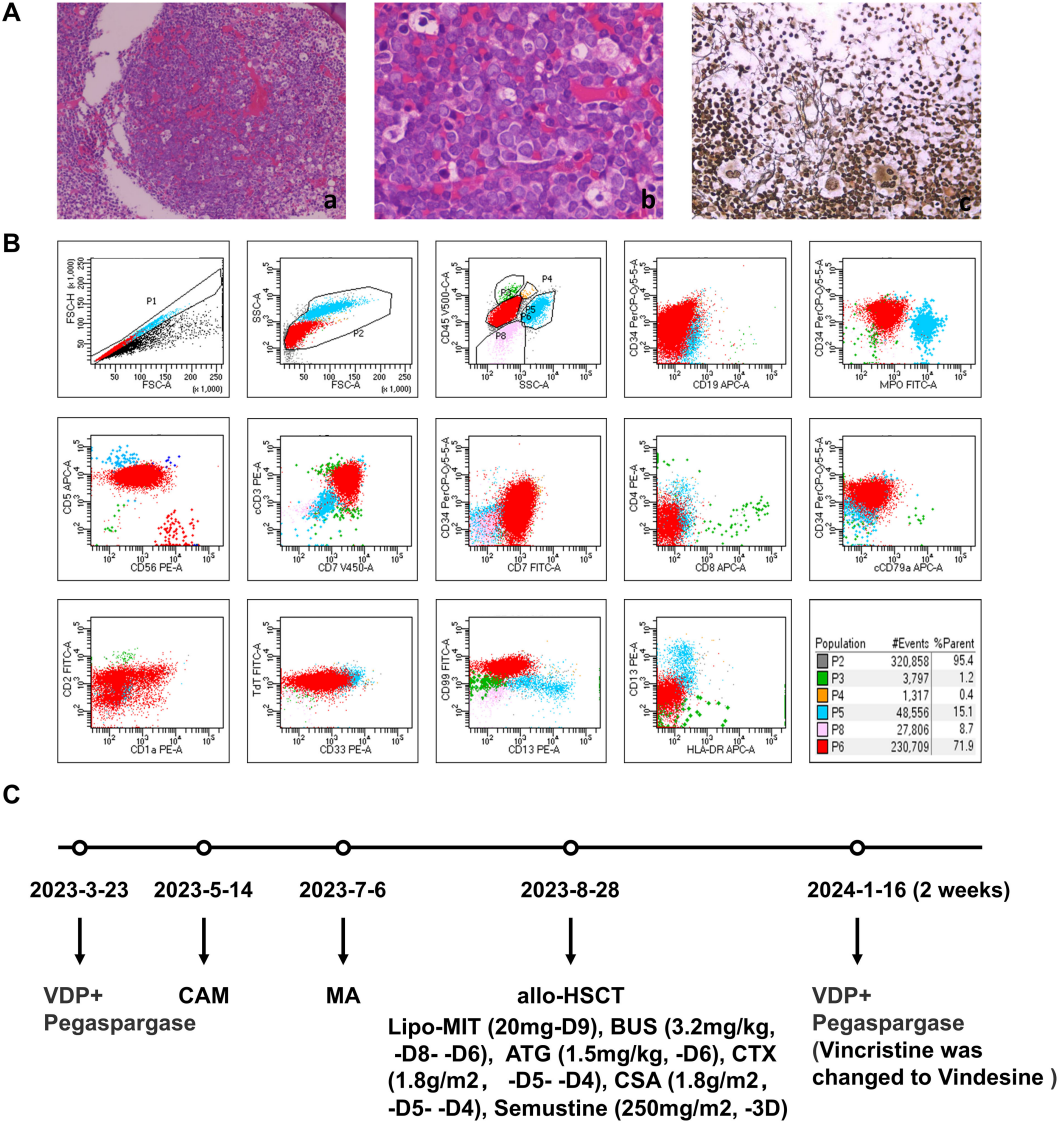
First diagnosis of T-ALL and the usage of drugs before CART therapy. **(A)** Bone marrow pathology. The area of nucleated cells was unevenly distributed, hyperplasia was extremely active in some areas, the original cells are diffuse, amd silver stain was positive. (a, b) hematoxylin and eosin stain. (c) silver stain. **(B)** Initial diagnosis of bone marrow MRD. 71.9% of nucleated cells expressed CD7, cCD3,CD5, CD38, TdT, some ceclls expressed CD34, CD1a, CD10, CD99, CD33, which did not express CD19,MPO,CD56. **(C)** The usage of drugs before CAR-T therapy. VDP, Vincristine, Daunorubicin, Dexamethasone; CAM, Cyclophosphamide, Cytarabine, Mercaptopurine; MA, Lipo-MIT, Cytarabine; Lipo-MIT, Mitoxantrone hydrochloride liposome; BUS, Busulfan; ATG, Anti-thymocyte Globulin; CSA, Cyclosporin A.

A 34-year-old male was admitted to the hematology department of our hospital for fatigue, dizziness, chest tightness. After completing the relevant examinations, he was diagnosed with T-cell acute lymphoblastic leukemia (T cells, KRAS, NOTCH1, PHF6 mutations) in March 2023. The diagnostic results were as follows: Bone marrow biopsy revealed malignant tumor (**Figure 1A**). Peripheral blood smears showed 66% primitive and naive cells. Bone marrow smear cytology showed hyperactive proliferation of primitive cells, and bone marrow flow immunotyping showed: 71.9% cells expressed CD7, CD34part, cCD3, CD5, CD38, CD1a part, CD10part, TDT, CD99 and CD33part (**Figure 1B**). Comprehensive detection of ALL/LBL related genes showed that KRAS variation abundance was 45.20%, NOTCH1 variation abundance was 39.5%, and PHF6 variation abundance was 93.5%.

The treatment plan was shown in **Figure 1C**. Bone marrow tests showed that complete remission (CR) was achieved after 2 weeks of treatment with VDP+PEG-asp (Vincristine, Daunorubicin, Dexamethasone and Pegaspargase) from March 23, 2023. Then, CAM (Cyclophosphamide, Cytarabine, and Mercaptopurine tablets), HD-MTX (methotrexate) and MA (Mitoxantrone hydrochloride liposome and Cytarabine) were used for consolidation chemotherapy. Allogeneic hematopoietic stem cell transplantation (allo-HSCT) pre-treatment (Mitoxantrone hydrochloride liposome, Busulfan, Cyclophosphamide, and Semustine) was performed from August 18, 2023. On August 28, 2023, a allogeneic hematopoietic stem cell transplantation of 170ml (CD34 3.15 × 10^6^/Kg, MNC 8.55 × 10^8^/Kg) was performed.

**Figure 2:**
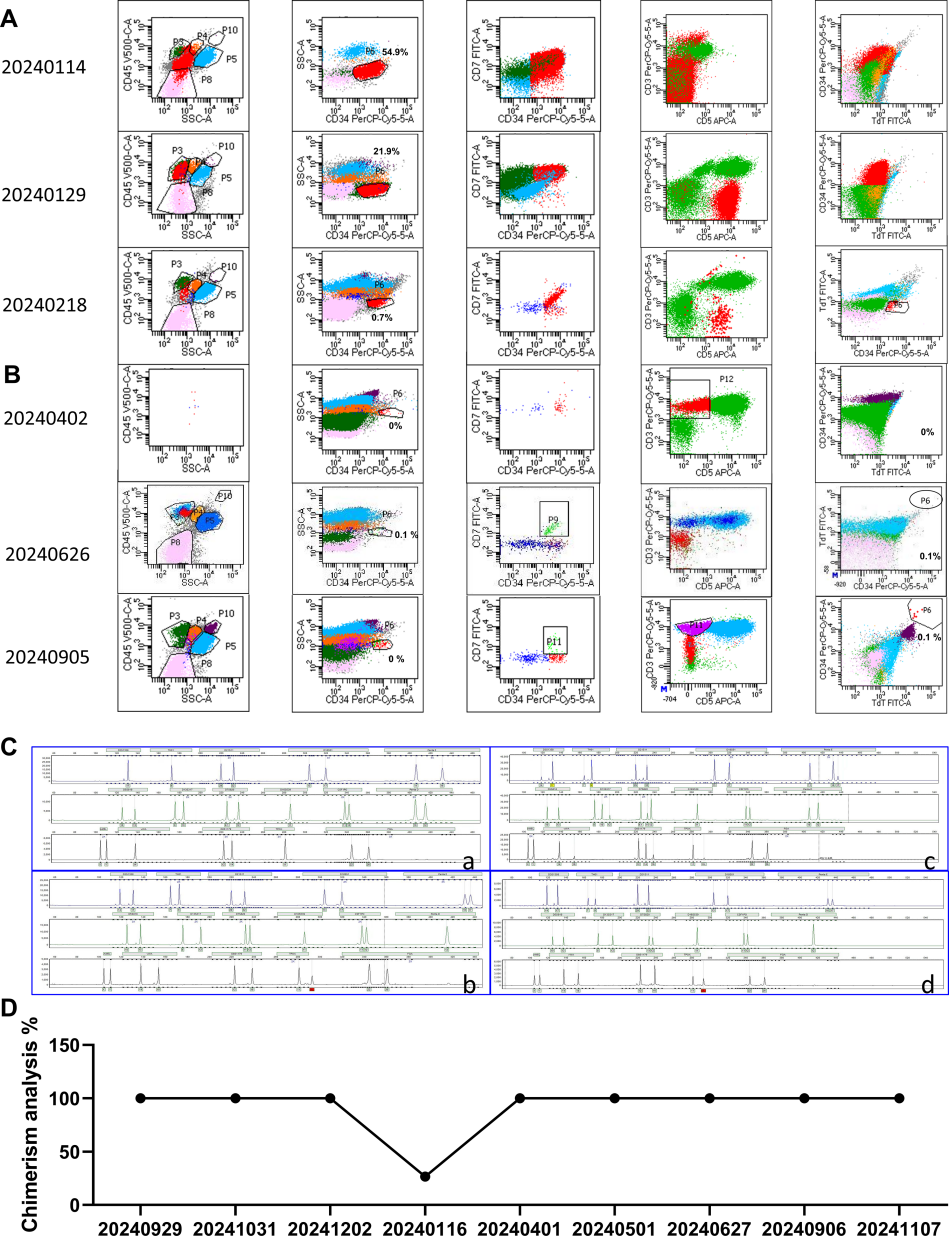
Bone marrow analysis. **(A)** Bone marrow MRD analysis before CAR-T therapy. **(B)** Bone marrow MRD analysis after CAR-T therapy. On day 26 after infusion, there were no abnormal naive T cells. **(C)** The fingerprint of the patient after allo-HSCT. (a) Pre-transplant fingerprint. (b) Post-transplant fingerprint (date: 20230929). (c) Fingerprint of relapse after Allo-HSCT treatment (date: 20240116). (d) Fingerprint of CR after CD7 CAR-T infusion (date: 20241107). **(D)** Changes in chimerism rate. The chimerism rate was 25.3% after relapse.

The patient was maintained CR for 4 months after allo-HSCT, and then he was diagnosed with a relapse of T-ALL based on peripheral blood smears showing a significant increase in the number of original cells, and bone marrow smears showing that the number of original cells accounted for 76.5%, minimal residual disease (MRD) results showed that 54.9% of the original cells were abnormal naive T cells (**Figure 2A**) and bone marrow chimerism decreased to 26.64% on January 15, 2024. After allo-HSCT, Cyclosporine was used for immunosuppression, and the concentration of Cyclosporine was monitored regularly to maintain the dose of 100-200ug/L, and immunosuppressive therapy was stopped immediately after relapse.

**Figure 3:**
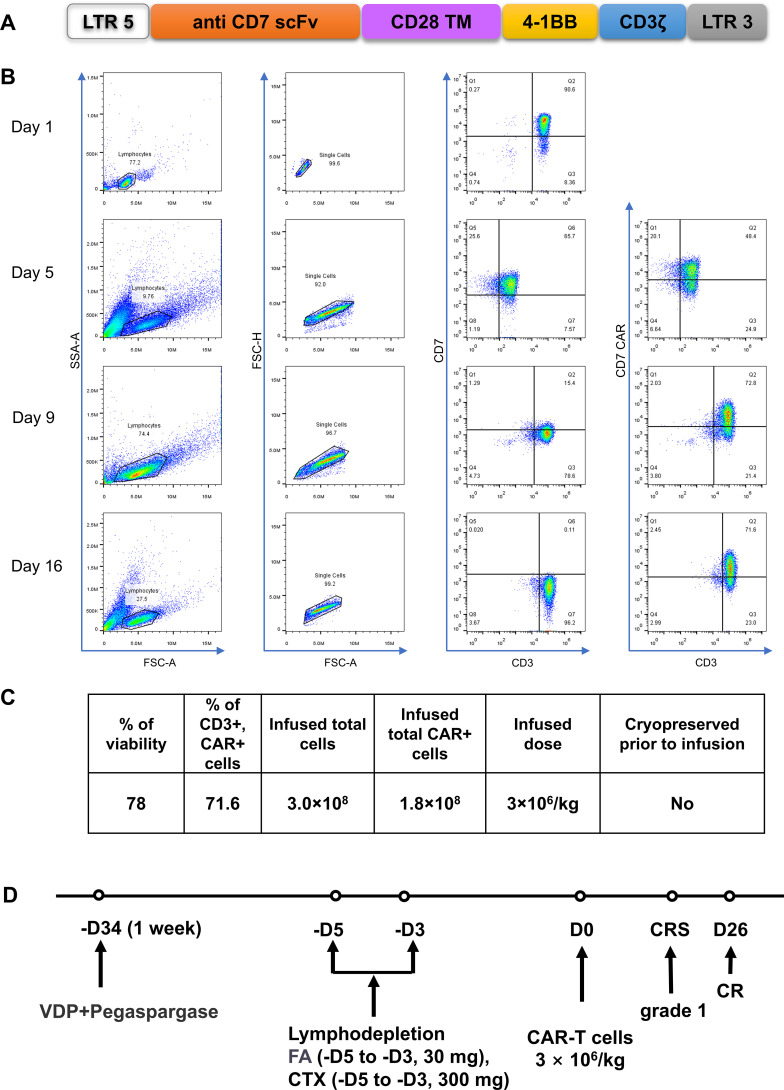
Construction of CD7-specific CAR and protocol of anti-CD7 CAR-T cell infusions. **(A)** a schematic diagram of anti-CD7 CAR vector. **(B)** Flow detection of CD7 and CD7 CAR during cells preparation. The proportion of CD7+T gradually decreased after RV infection. **(C)** Characterization of the final anti-CD7 CAR-T cell product. VDP, Vindesine, Lipo-MIT, Dexamethasone; FA, Fludarabine; CTX, Cyclophosphamide. **(D)** a protocol of Treatment. FC for lymphodepletion, FA(30 mg, -D5~-D3) and CTX (300 mg, -D5~-D3). CAR-T cells were infused at a dose of 3×106/kg.

After 2 weeks of treatment with VDP (Vindesine, Daunorubicin, Dexamethasone) +PEG asp, bone marrow smears showed that primitive immature cells accounted for 63.5%, and minimal residual disease (MRD) results showed that 21.9% of original cells were abnormal naive T cells (**Figure 2A**). Based on the unsatisfactory chemotherapy effect mentioned above, it was considered to change the chemotherapy regimen and combine CAR-T cells for treatment. Before immune cell therapy, VDP (Daunorubicin was changed to Mitoxantrone hydrochloride liposome) +PEG asp chemotherapy regimen was used. After one week of treatment, although the flow cytometry results on February 18, 2024 showed an MRD of 0.7% (**Figure 2A**), considering the high risk of disease recurrence and progression, subsequent infusion of allogeneic CAR-T cells aims to improve efficacy. The structure of the CAR is shown in **Figure 3A**. PBMCs were collected at D-16 for CAR-T cell preparation. After 16 days culture *in vitro*, the proportion of CD7+ T cells continued to decrease (**Figure 3B**), indicating that CD7 CAR-T exerted a killing effect on CD7+ T cells. We also performed killing assays to demonstrate the efficacy of CD7 CAR-T before infusion *in vitro* (**Supplementary Figure 1**). Fludarabine and Cyclophosphamide were used for lymphodepletion on March 01, 2024, then 50mL (total 1.80×10^8^ cells) anti-CD7 CAR-T infusion was performed on March 06, 2024 (**Figures 3C, D**). During the treatment, the patient took oral probiotics to maintain intestinal flora homeostasis and prevent Graft versus host disease (GVHD), and then probiotics were administered long-term.

**Figure 4:**
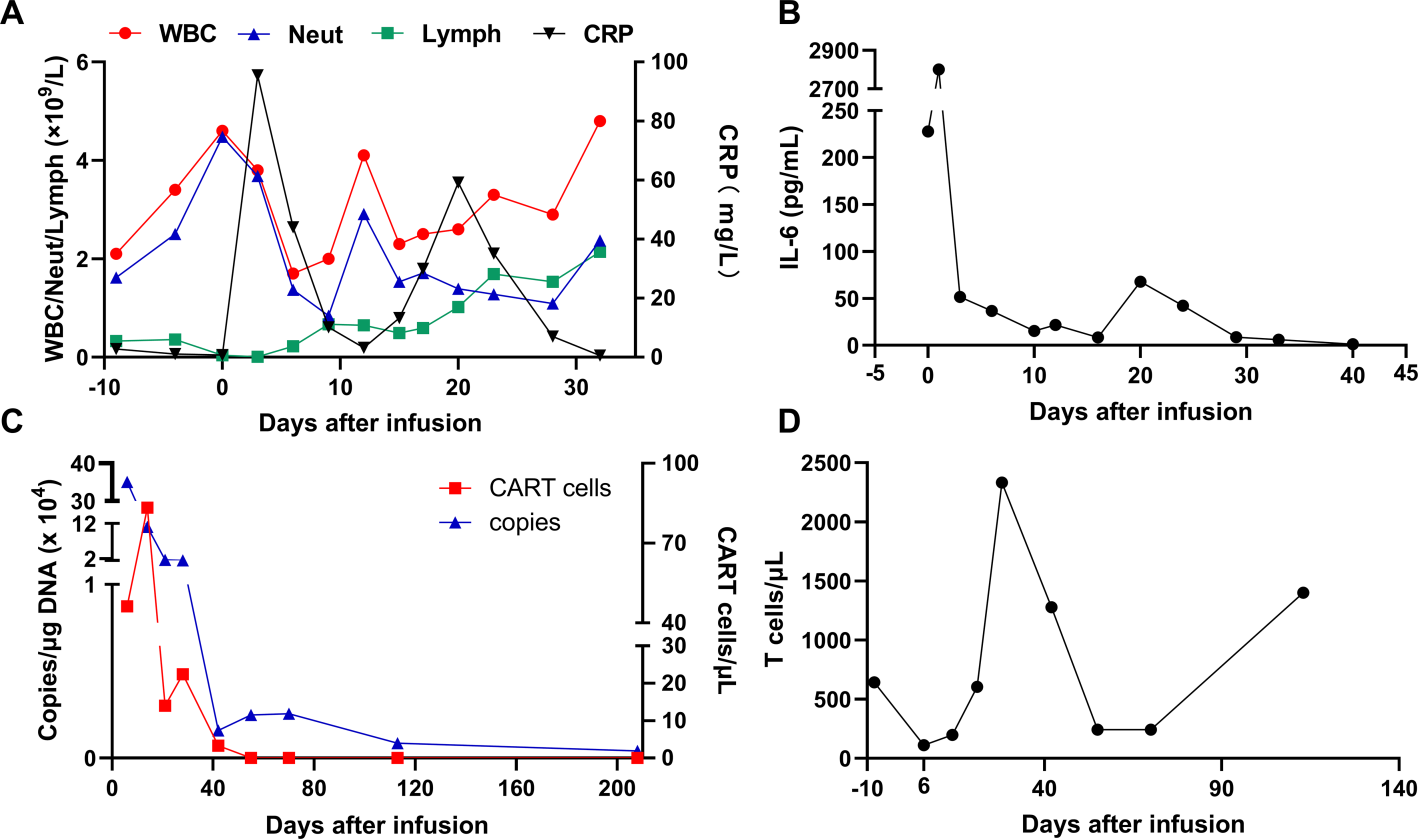
Clinical responses and levels of key indicators to infusions of anti-CD7 CAR-T cells. **(A)** The changes of patient’s WBC, Neut, Lymph, CRP. **(B)** Levels of IL-6 after CAR-T cell infusion. IL-6 peaked on the 1st day (2801 pg/mL) and returned to normal levels on the 12th day. **(C)** CAR-T cell expansion levels detected by flow cytometry and CAR DNA copies detected by qPCR. CAR-T peaked on day 14 and CAR DNA copies peaked on day 6 after infusion (CD3+ CAR+T cells: 83 cells/μL, CAR DNA copies:350758 copies/μg). **(D)** T cell counts analysis. T cell counts (111.8 cells/μL) were lowest on day 6 after infusion.

Two days after the infusion of anti-CD7 CAR-T, the patient developed severe neutropenia with fever and a maximum temperature of 39.0°C on March 07, 2024. At this time, the count of patient’s WBC and Neut decreased (**Figure 4A**), the levels of serum CRP (**Figure 4A**) and IL-6 (**Figure 4B**) also increased accordingly, which reached the highest peak within 1 week after infusion. Patients were considered to have grade 1 CRS according to the guidelines of the CARTOX Working group (13). Ibuprofen was used for symptomatic treatment, Cefoperazone sulbactam sodium or meropenem or tigecycline were used for anti-infective treatment depending on the patient, and γ-Globulin was intermittently supplemented to enhance immunity. The RV copy numbers of CD7-CARs reached the peak at 350, 758 copies/μg by using qPCR to quantitatively analyse (**Figure 4C**). After one week infusion, a decrease in the proportion of T cells was followed by an increase (**Figure 4D**), indicating that CD7 CAR-T exerted effect for T-ALL. According to the bone marrow MRD analysis of T-ALL, MRD remained negative from the 26th day after CAR-T infusion (**Figure 2B**; **Supplementary Figure 2**), indicating that the patient’s condition was effectively controlled and achieved CR. At the same time, the bone marrow chimerism rate recovered from 26.64% before infusion to 100% (**Figures 2C, D**) and the patient did not experience any symptoms related to GVHD, such as gut microbiota disruption caused by diarrhea (**Supplementary Figure 3**).

After CAR-T therapy and symptomatic treatment, the patient’s condition improved and MRD was continuously negative, which was been 9 months without recurrence.

## Discussion

CD7 is a transmembrane glycoprotein expressed in 90% -96% of normal T cells (14), and is a potential target for the treatment of T-cell lymphoma. CAR-T cells targeting CD7 are considered to have a high CR rate in T-ALL (15, 16). For T-LBL with bone marrow involvement, autologous CD7 CAR-T cell therapy is currently not feasible due to the presence of a common antigen between normal and malignant T cells. The patient did not achieve satisfactory results after undergoing allo-HSCT treatment in this case. In recent years, allo-HSCT combined with CAR-T syndrome has gradually become an effective treatment measure (16). Studies have shown that patients receiving CD7 CAR-T and sequential allo-HSCT have achieved satisfactory therapeutic effects (16, 17). However, pretreatment and allo-HSCT may also lead to depletion of CAR-T cells *in vivo*, affecting the long-term efficacy of CAR-T cell therapy (17). How to further optimize the CAR-T combined with allo-HSCT strategy has become a problem that researchers need to solve. In this case, after allogeneic hematopoietic stem cell transplantation, the patient further underwent chemotherapy to reduce tumor burden and received donor derived CD7 CAR-T infusion to achieve CR efficacy. After administering CD7 CAR-T from the donor source, the patient did not experience any GVHD related symptoms such as diarrhea. It was considered that using gut microbiota as adjuvant therapy could alleviate the symptoms of GVHD (18, 19).

As a key factor in the preparation of CAR-T cells, the design of gene transduction vectors is currently mainly based on the use of RV and lentiviral vectors (20, 21). However, compared with lentiviral vectors, RV exhibits advantages in industrial production due to its ability to generate stable toxin producing cell lines, low plasmid dosage, low impurity content, good transduction effect, and high viral vector titer. So as to meet the requirements of high-throughput and low-cost CAR-T production, while ensuring effectiveness and safety (22, 23). Therefore, in this case, the novel anti CD7 CAR-T cells were prepared using RV. Not only has it achieved significant clinical therapeutic effects, but it also demonstrates the feasibility and cost control advantages of RV application in CAR-T cell production. Meanwhile, CAR-T cells did not exhibit abnormal proliferation. These results demonstrate that RV produced anti CD7 CAR-T cells have good clinical efficacy and safety.

## Conclusion

In summary, this patient received allo-HSCT support, followed by chemotherapy and sequential anti-CD7 CAR-T cell immunotherapy. On the one hand, chemotherapy was used to reduce the burden of tumors, thereby lowering the risk of CRS response. On the other hand, it reflected the advantage of CAR-T cells in further precise tumor clearance *in vivo*, and once again validated the advantage of RV in preparing CAR-T cells to control tumor treatment costs, which also provided an effective clinical product for the treatment of relapsed/refractory T-ALL. This case demonstrated that RV-based anti-CD7 CAR-T cells have good therapeutic effects on patients with relapsed/refractory T-ALL. This is also the first clinical case of using RV to prepare anti-CD7 CAR-T cells.

## Data Availability

The original contributions presented in the study are included in the article/**Supplementary Material**. Further inquiries can be directed to the corresponding authors.

## Supplementary material

Supplementary Figure 1 The killing activity of CD7 CAR-T was detected by flow cytometry. Anti-CD7 CAR-T cells were co-cultured with Jurkat cells at a gradient of E/T ratio for 12 hours. CFSE and Annexin V were measured.

Supplementary Figure 2 Bone marrow MRD analysis for November 7, 2024. Phenotypically abnormal primitive/naive T lymphocytes were not detected by flow cytometry in bone marrow samples. Mature T cells accounted for about 38.9% of the total number of nuclear cells, and their immunophenotype was CD3+, CD5+, CD2+, CD7 deficient expression, CD34-, CD99-, nTdT-, cCD3+.

Supplementary Figure 3 Changes of intestinal flora after CAR-T infusion. **(A)** The relative abundance of fecal intestinal flora before and after CD7 CAR-T infusion at the gate level. **(A)** Relative abundance of beneficial and harmful bacteria in intestine before and after after CD7 CAR-T infusion. **(a)** Before infusion. **(b)** After infusion.
