# Supplementary figures and images for: Case report: The case of T-cell acute lymphoblastic leukemia treated with chemotherapy followed by anti-CD7 CAR-T cells using retroviral vector
Source: Front Immunol. 2025 Jan 14;15:1519055. doi: 10.3389/fimmu.2024.1519055 (PMC11772494; doi:10.3389/fimmu.2024.1519055)

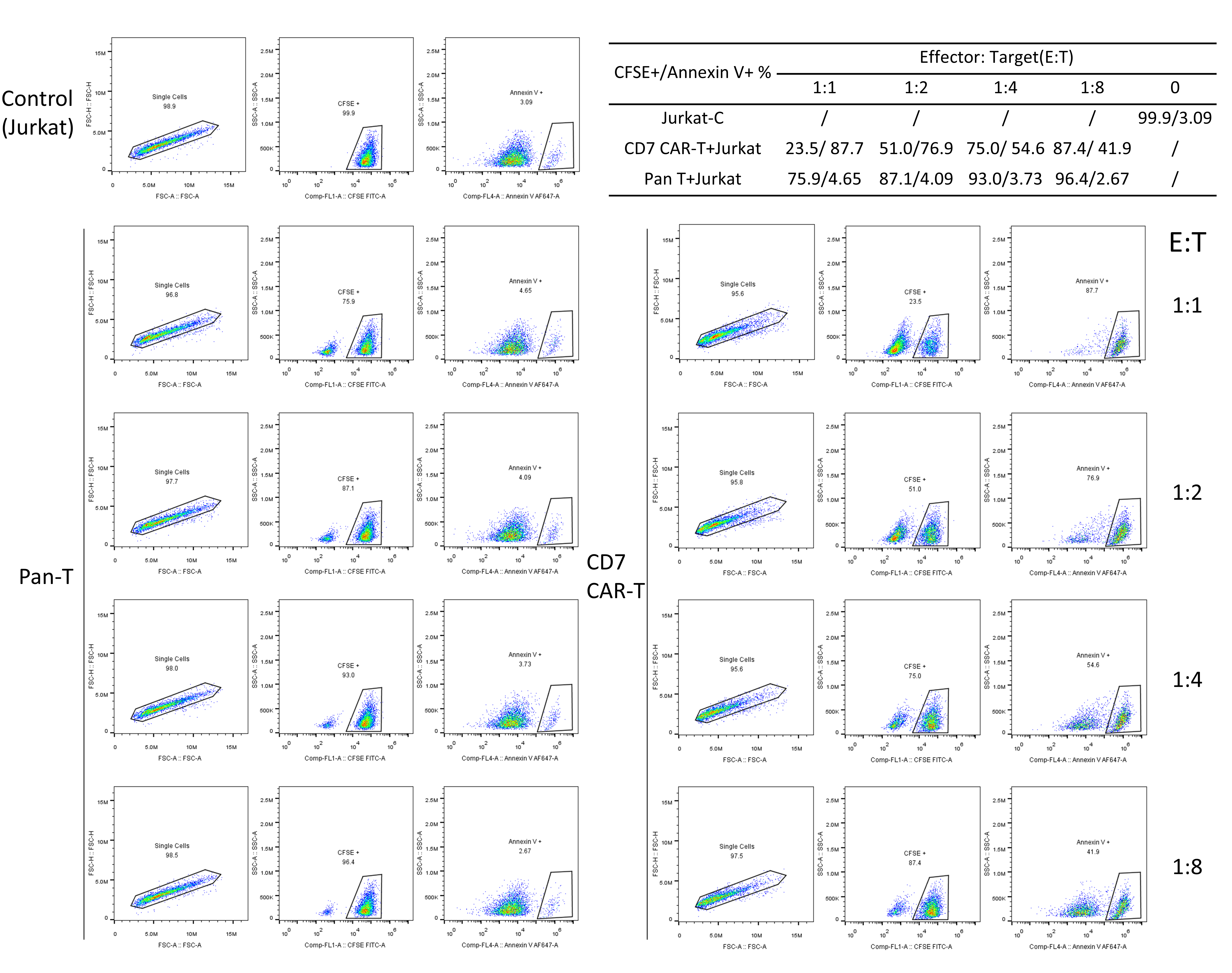

Supplement: Supplementary Figure 1 — The killing activity of CD7 CAR-T was detected by flow cytometry. Anti-CD7 CAR-T cells were co-cultured with Jurkat cells at a gradient of E/T ratio for 12 hours. CFSE and Annexin V were measured. [file Image1.tif]

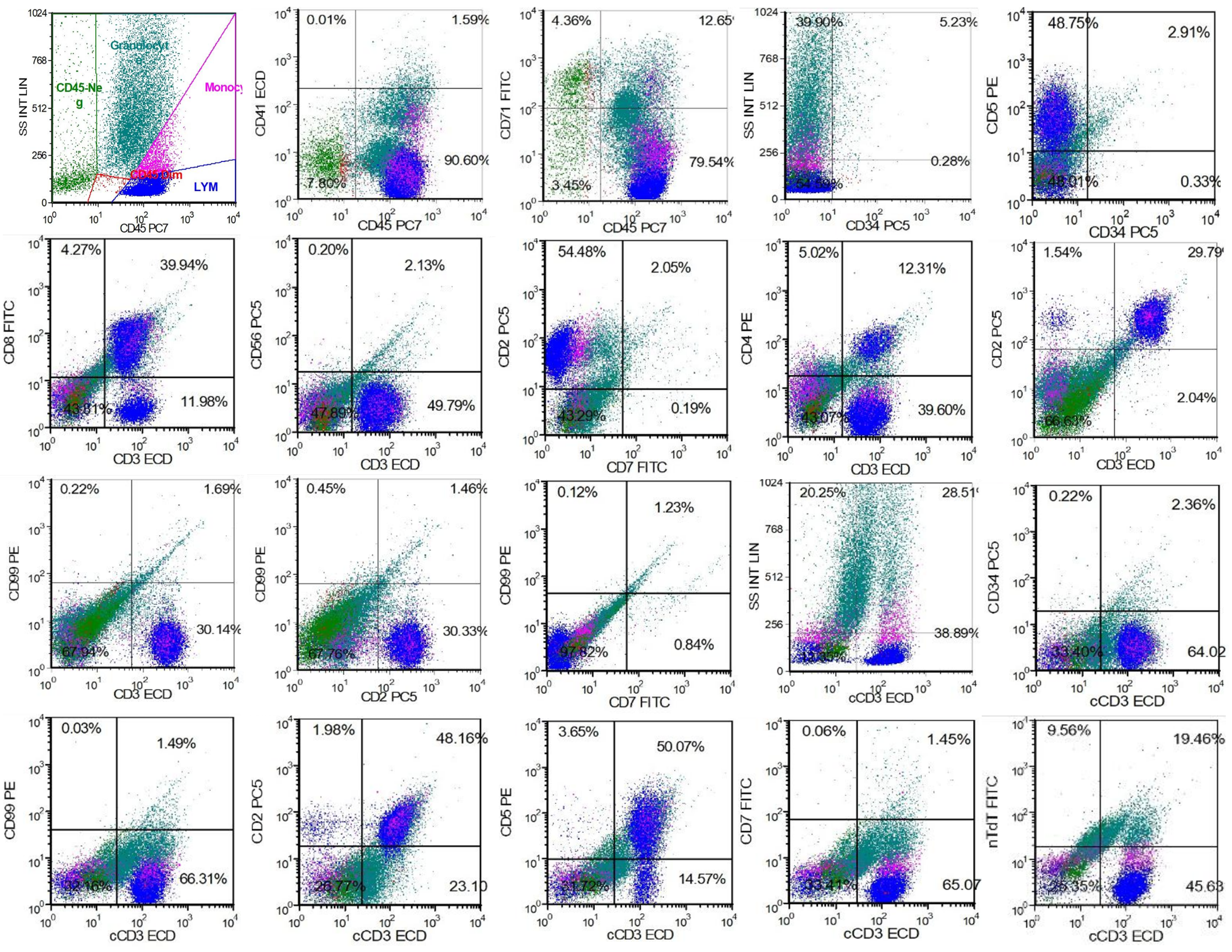

Supplement: Supplementary Figure 2 — Bone marrow MRD analysis for November 7, 2024. Phenotypically abnormal primitive/naive T lymphocytes were not detected by flow cytometry in bone marrow samples. Mature T cells accounted for about 38.9% of the total number of nuclear cells, and their immunophenotype was CD3+, CD5+, CD2+, CD7 deficient expression, CD34-, CD99-, nTdT-, cCD3+. [file Image2.tif]

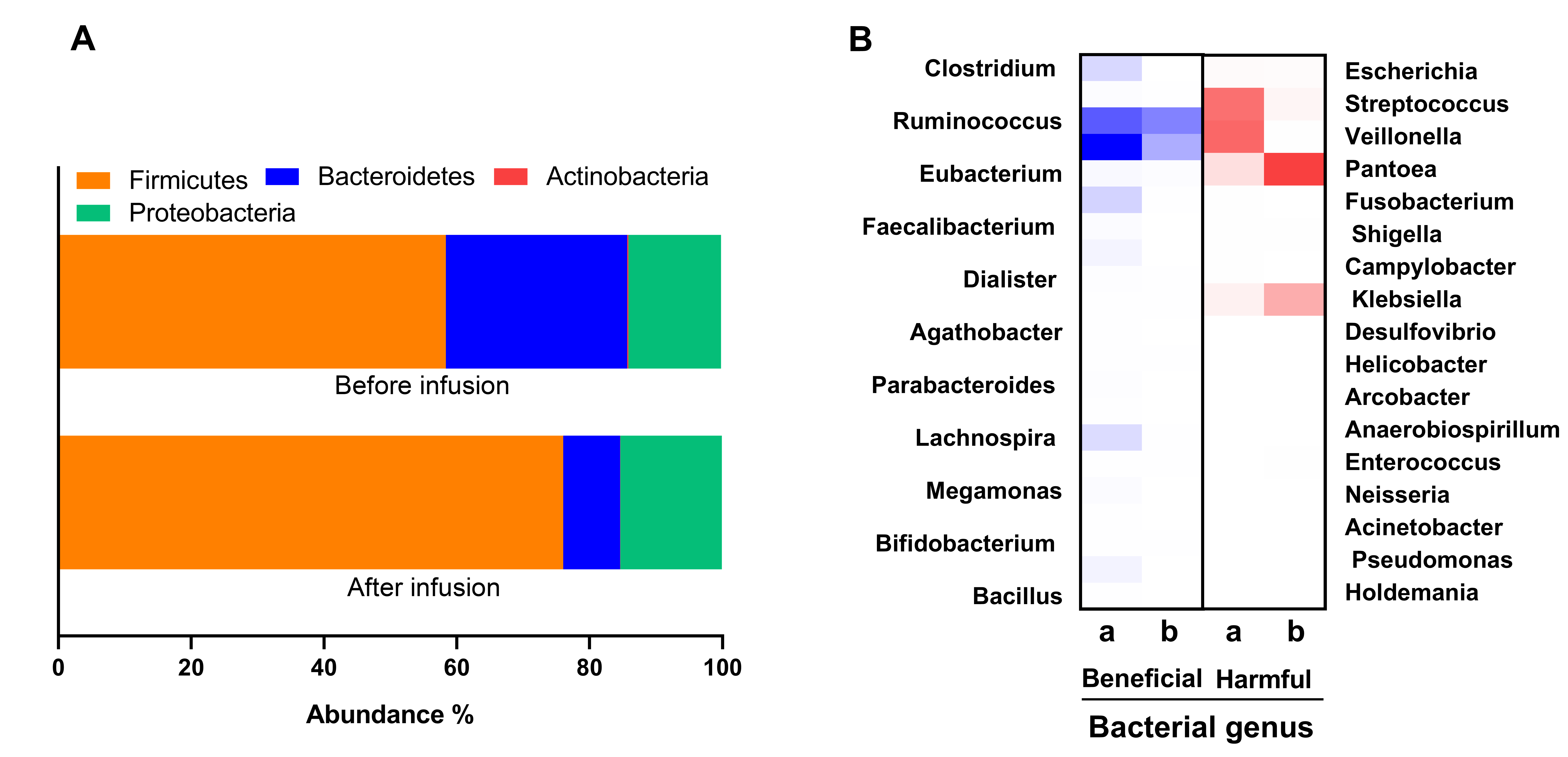

Supplement: Supplementary Figure 3 — Changes of intestinal flora after CAR-T infusion. (A) The relative abundance of fecal intestinal flora before and after CD7 CAR-T infusion at the gate level. (A) Relative abundance of beneficial and harmful bacteria in intestine before and after after CD7 CAR-T infusion. (a) Before infusion. (b) After infusion. [file Image3.tif]
